# Trends in Medicare Spending and Utilization of Oral Medications for *Clostridioides difficile* Infection From 2013 to 2021

**DOI:** 10.1016/j.gastha.2023.11.010

**Published:** 2023-11-23

**Authors:** Xiaohan Ying, Lasha Gogokhia, Arun B. Jesudian, Lillian Zhang, Carl V. Crawford

**Affiliations:** 1 Department of Internal Medicine, Weill Cornell Medicine, New York, New York; 2 Division of Gastroenterology and Hepatology, Weill Cornell Medicine, New York, New York

*Clostridioides difficile* infection (CDI) is a major cause of antibiotic-associated diarrhea, and its prevalence has significantly increased with the widespread use of antibiotics. In the United States, CDI is estimated to infect approximately 224,000 patients annually, causing over 12,800 deaths a year.^1^ While the incidence of healthcare–associated CDI has decreased over past several years, owing to improved hospital infection control and antibiotic stewardship, there has been an increase in community associated infections.

In 2011, the Food and Drug Administration approved Dificid®(fidaxomicin), a macrocyclic antibiotic, for the treatment of CDI. Phase III trials published in the New England Journal of Medicine and the Lancet Infectious Diseases showed that fidaxomicin significantly reduced rates of recurrent CDI while providing similar safety profiles.^2^^,^^3^ Additional studies have also found fidaxomicin to have superior sustained clinical response and prevention of recurrent CDI.^4^^,^^5^ Despite more favorable outcomes of using fidaxomicin for the treatment of CDI, its use has been limited largely due to high cost.

In this study, we aim to analyze trends of Medicare Part D utilization and spending on medications for CDI.

In this cross-sectional study, we used publicly available Medicare Part D data (2013–2021) to determine the number of beneficiaries receiving these drugs and the total associated annual spending. Four medications were included in this study: Vancocin® (vancomycin HCl capsule), Firvanq® (vancomycin HCl oral solution), generic vancomycin HCl, and fidaxomicin. The JoinPoint Regression Program (version 4.9.1.0; National Cancer Institute, Bethesda, MD) was used for annual percent change (APC) and trends analysis. We followed the STROBE practice guidelines.

Between 2013 and 2021, total Medicare part D spending on oral CDI medications was $1.34 billion; 74.9% ($1.0 billion) was for generic vancomycin HCl, followed by 23.8% ($319.4 million) for fidaxomicin, Firvanq® (0.7%), and Vancocin® (0.7%). Figure A However, in terms of number of beneficiaries receiving these medications, generic vancomycin HCl accounted for 93.0% (1.3 million), compared to 4.6% (65,721) for fidaxomicin. Figure B.

**Figure:**
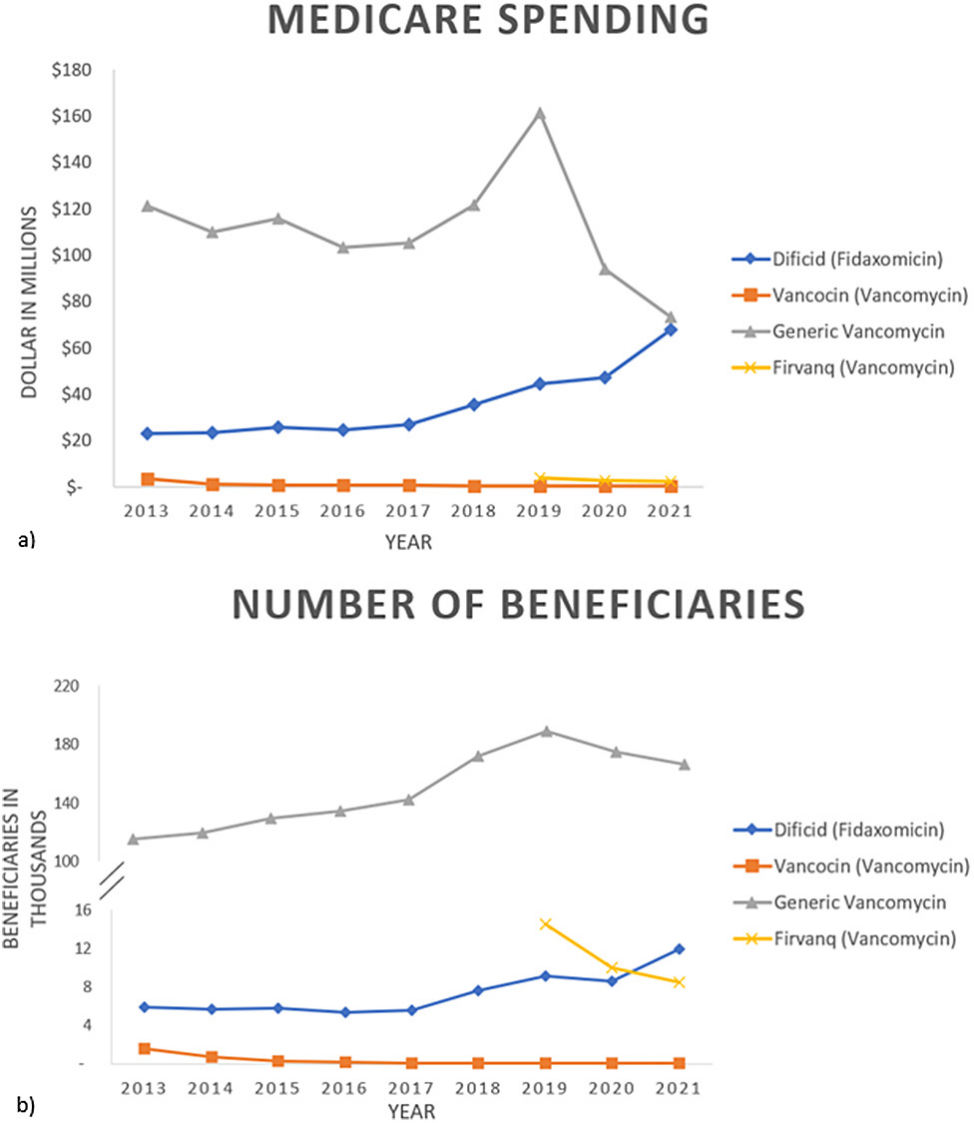
Total Medicare Spending and Utilization of oral CDI Medications. (A) Medicare Spending on Oral CDI Medications; (B) Number of Medicare Beneficiaries Receiving Oral CDI Medications.

Medicare spending of fidaxomicin was fairly stable from 2013 and 2017, after which it experienced a significant increase from $27.0 million in $2017 to $67.9 million in 2021 (2013–2017: APC 4.25, 95% confidence interval [CI]: −4.3 to 13.6, *P* = .248; 2017–2021: APC 24.6, 95% CI: 14.4 to 35.7, *P* = .002). A similar trend was shown for the number of beneficiaries who received this medication (2013–2016: APC -3.5, 95% CI: −20.7 to 17.4, *P* = .638; 2016–2021: APC 16.4, 95% CI: 6.6 to 27.0, *P* = .009). Lastly, per beneficiary spending experienced a steady increase since 2013, from $3592 per person in 2013 to $5678 per person in 2021 (APC 4.3, 95% CI: 3.2 to 5.4, *P* < .001).

Generic vancomycin HCl accounted for over 98% of total Medicare spending for oral vancomycin, whereas Vancocin® and Firvanq® accounted for less than 1% each. Medicare spending for generic oral vancomycin HCl experienced an upward trend from 2013 to 2019, and a downward trend since 2019, although neither was statistically significant (2013–2019: APC 3.3, 95% CI: −1.8 to 8.8, *P* = .151; 2019–2021: APC -25.6, 95% CI: −45.1 to 0.8, *P* = .054). Total number of beneficiaries increased from 115,119 in 2013 to 188,576 in 2019 (2013–2019: APC 8.6, 95% CI: 5.2 to 12.2, *P* = .002, 2019–2021: APC – 3.9, 95% CI: −20.5 to 16.2, *P* = .595). The per person cost for generic vancomycin HCl experienced a steady decline from $1053 in 2013 to $441 in 2021 (APC -8.4, 95% CI: −12.3 to −4.4, *P* = .002).

Our study showed that since 2016, fidaxomicin has experienced a significant increase in its utilization within the Medicare population despite its higher cost. This is likely due to 1) growing body of evidence favoring fidaxomicin in terms of clinical outcomes and overall cost savings when compared to vancomycin for the treatment of CDI, and 2) fidaxomicin being included in the Infectious Diseases Society of America (IDSA). One study published in Clinical Microbiology and Infection concluded that fidaxomicin achieved a higher level of quality adjusted life years gain at a lower cost.^6^ Additional studies have reached similar conclusions; despite higher initial acquisition cost of fidaxomicin, that overall cost of treatment was offset by lower total hospitalization costs, lower costs of managing adverse events, and lower incidence of CDI recurrence and adverse events.^7^^,^^8^ Fidaxomicin and vancomycin were both recommended as first line treatment for CDI per IDSA’s 2017 guideline.^9^ In 2021, IDSA and Society for Healthcare Epidemiology of America recommended fidaxomicin rather than vancomycin as first line treatment for initial and recurrent CDI due to its improved sustained clinical response and lower risk of CDI recurrence.^10^

However, despite its recent increase in prescriptions, the number of Medicare beneficiaries who received fidaxomicin accounted for less than 10% of the number of beneficiaries who received vancomycin. This could be due to both provider (eg slower rate of adoption, concerns with cost of medications) and patient (eg higher out-of-pocket cost for fidaxomicin) factors.

Our study has several limitations. Firstly, Medicare Part D database does not report patient characteristics or other clinical variables (eg age, comorbidities, prior antibiotics use, etc.), which prevents us to perform further in depth studies on variables that could possibly confound prescription patterns. Secondly, oral metronidazole was not included in this study despite still being used for the treatment of CDI in some practices. This is because the database does not include treatment indication or route of administration for metronidazole.

In conclusion, there has been a significant increase in the uptake of fidaxomicin, which has been shown to have higher sustained clinical cure rates, lower recurrence rates, and provides overall cost savings for patients with CDI. Further studies should be performed to better understand utilization patterns for fidaxomicin since the updated IDSA and Society for Healthcare Epidemiology of America guidelines.
